# Machine learning-based dynamic prediction of lateral lymph node metastasis in patients with papillary thyroid cancer

**DOI:** 10.3389/fendo.2022.1019037

**Published:** 2022-10-10

**Authors:** Sheng-wei Lai, Yun-long Fan, Yu-hua Zhu, Fei Zhang, Zheng Guo, Bing Wang, Zheng Wan, Pei-lin Liu, Ning Yu, Han-dai Qin

**Affiliations:** ^1^ Medical School of Chinese PLA, Beijing, China; ^2^ Department of Otolaryngology Head and Neck Surgery, The First Medical Centre of Chinese PLA General Hospital, Beijing, China; ^3^ Department of General Surgery, The First Medical Centre of Chinese PLA General Hospital, Beijing, China; ^4^ The Third Team, Academy of Basic Medicine, The Fourth Military Medical University, Xi’an, China

**Keywords:** machine learning, central lymph node metastasis, papillary thyroid cancer, feature selection, model interpretation, dynamic prediction

## Abstract

**Objective:**

To develop a web-based machine learning server to predict lateral lymph node metastasis (LLNM) in papillary thyroid cancer (PTC) patients.

**Methods:**

Clinical data for PTC patients who underwent primary thyroidectomy at our hospital between January 2015 and December 2020, with pathologically confirmed presence or absence of any LLNM finding, were retrospectively reviewed. We built all models from a training set (80%) and assessed them in a test set (20%), using algorithms including decision tree, XGBoost, random forest, support vector machine, neural network, and K-nearest neighbor algorithm. Their performance was measured against a previously established nomogram using area under the receiver operating characteristic curve (AUC), decision curve analysis (DCA), precision, recall, accuracy, F1 score, specificity, and sensitivity. Interpretable machine learning was used for identifying potential relationships between variables and LLNM, and a web-based tool was created for use by clinicians.

**Results:**

A total of 1135 (62.53%) out of 1815 PTC patients enrolled in this study experienced LLNM episodes. In predicting LLNM, the best algorithm was random forest. In determining feature importance, the AUC reached 0.80, with an accuracy of 0.74, sensitivity of 0.89, and F1 score of 0.81. In addition, DCA showed that random forest held a higher clinical net benefit. Random forest identified tumor size, lymph node microcalcification, age, lymph node size, and tumor location as the most influentials in predicting LLNM. And the website tool is freely accessible at http://43.138.62.202/.

**Conclusion:**

The results showed that machine learning can be used to enable accurate prediction for LLNM in PTC patients, and that the web tool allowed for LLNM risk assessment at the individual level.

## Introduction

Over the past few decades, thyroid cancer has been steadily on the rise worldwide (1). With increasing social awareness of the disease, more cases of early-stage thyroid cancer are being screened and treated, particularly papillary thyroid cancer (PTC), the most common type of pathology, accounting for approximately 85% to 90% of all cases (2, 3). PTC patients are usually associated with a fairly good prognosis. With standard surgery and adjuvant radioiodine therapy, the 10-year survival rate for PTC could achieve 97% (4). However, lateral lymph node metastases (LLNM) were found in 18% to 64% of patients (5). Notably, there are evidences that LLNM is an independent risk factor associated with cancer recurrence and poor disease-free survival, and some patients may develop local invasion and treatment resistance (6).

Clinically, preoperative ultrasonography and computed tomography for screening suspected cervical LLNM are highly specific but of low sensitivity, particularly in the evaluation of occult LLNM, which are of limited value (7). Lateral lymph node dissection is not recommended unless suspicious LLNM is confirmed by preoperative imaging and fine needle aspiration biopsy (FNAB). As a result, timing and quality of care may suffer, for many patients who undergo thyroidectomy may still be left with LLNM after surgery (8). Lateral lymph node dissection is associated with complications such as hypoparathyroidism, neck pain, and chyle leakage, with a much higher complication rate than non-dissection procedures. Given this, a reasonable lateral lymph node dissection strategy during surgery is important, as excessive or inadequate dissection could bring about considerable impact on patient outcomes (9). For research and clinical practice, it would therefore be of eminent importance to develop a reliably predictive model to monitor LLNM.

Machine learning is a new computer-based data analysis method now being widely used in the medical field, especially in radiology, ophthalmology, and dermatology (10). Compared to traditional statistical methods such as logistic regression, machine learning enables more interactions between variables and outcomes to be found. However, to our knowledge, studies on employing machine learning for predicting LLNM in PTC patients are still absent (11). In fact, establishing a robust predictive model for PTC would help clinicians stratify high-risk patients for intensive treatment and propose candidates for active follow-up. In the present study, we proposed a machine learning-based model for predicting LLNM in a preoperative context and identifying risk factors associated with LLNM in patients with PTC. Specifically, a website tool was generated to allow clinical use, and the proposed model was subjected to critical evaluation.

## Methods

### Study population

**Figure 1:**
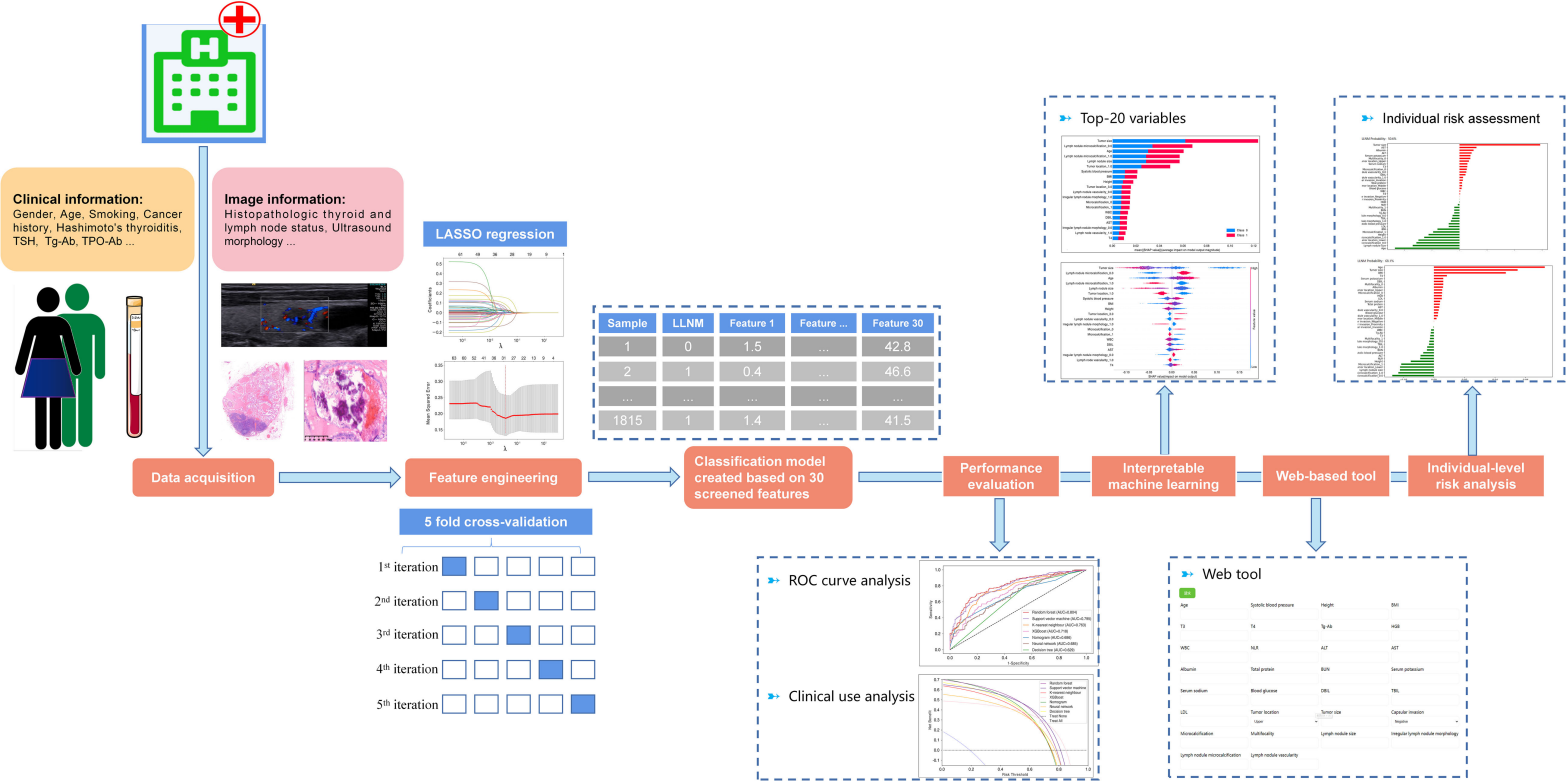
Overview of the analysis workflow.

Data on patients who underwent thyroidectomy between January 2015 and December 2020 were extracted from the electronic health records of one medical center, the First Medical Centre of the Chinese PLA General Hospital, for analysis. With approval from the Institutional Review Board of the Chinese PLA General Hospital, the study was exempt from informed consent due to its retrospective research nature. The study was reported following the recommendations of the Transparent Reporting of prediction model development and validation for Individual Prognosis Or Diagnosis (TRIPOD) statement. Our analysis workflow is presented in **Figure 1**.

**Figure 2:**
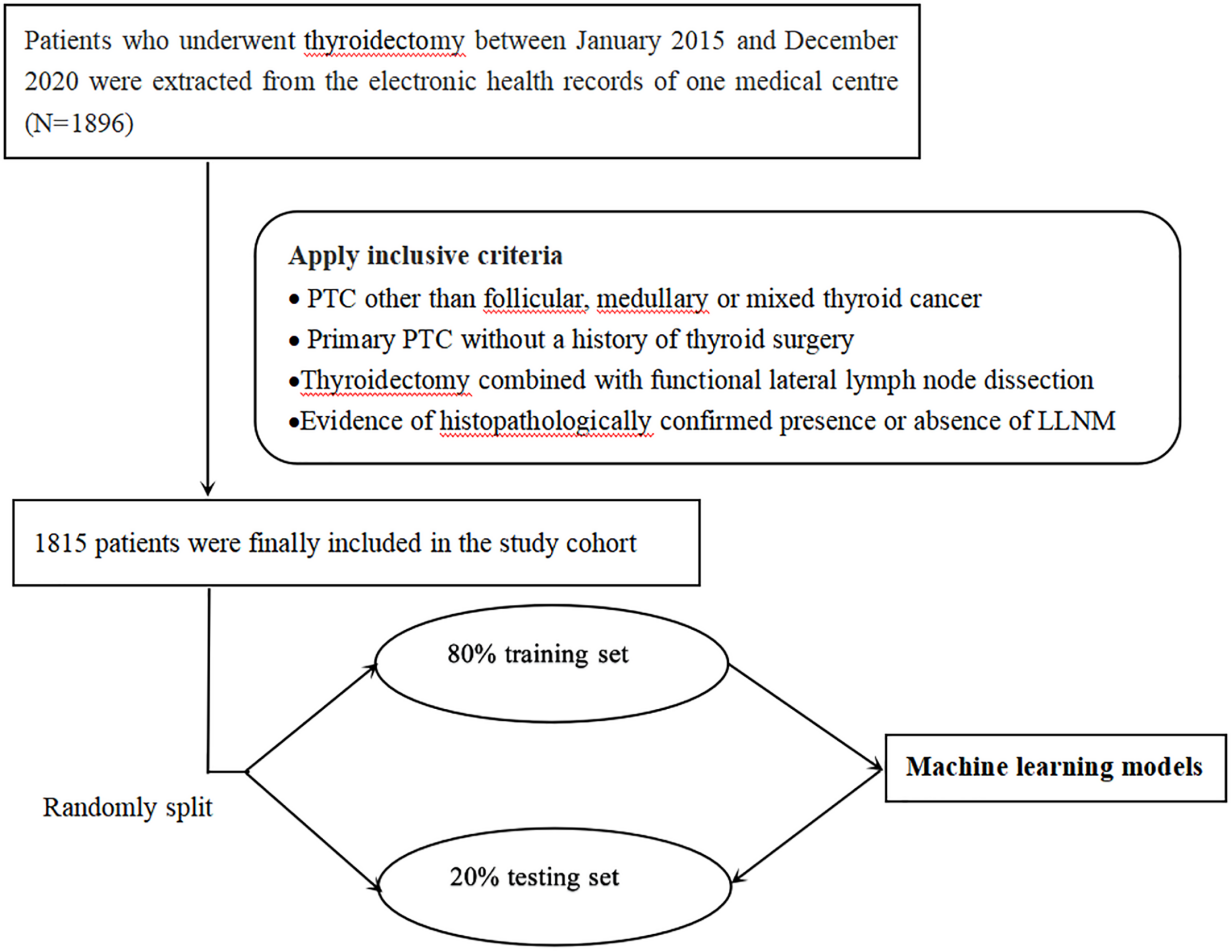
Flow chart of patient selection.

The surgical decision-making process followed the 2015 American Thyroid Association (ATA) guidelines. Notably, for better generalization to real-world clinical practice, the study adopted broad inclusion criteria and minimal exclusion criteria. The inclusion criteria were as follows. (1) PTC other than follicular, medullary, or mixed thyroid cancer; (2) primary PTC without a history of thyroid surgery; (3) thyroidectomy with unilateral or bilateral central lymph node dissection, combined with functional lateral lymph node dissection; and (4) evidence of histopathologically confirmed presence or absence of LLNM. Finally, a total of 1815 patients were screened for model development in this study, including 1135 who suffered LLNM and 680 who did not (**Figure 2**).

### Data acquisition

Information on the clinical characteristics, laboratory findings, and ultrasound features of the patients were retrospectively collected for analysis. Clinical characteristics included gender, age, height, weight, body mass index, smoking, alcohol, menopause, hypertension, diabetes mellitus, dyslipidemia, personal cancer history, family thyroid cancer history, family other cancer history, systolic blood pressure, diastolic blood pressure, and mean arterial blood pressure. Thyroid function tests on the laboratory findings covered triiodothyronine (T3), tetraiodothyronine (T4), free T3 (FT3), free T4 (FT4), thyroid-stimulating hormone (TSH), anti-thyroglobulin antibody (Tg-Ab), and anti-thyroid peroxidase antibody (TPO-Ab). Based on the ultrasound, the following features were recorded: tumor size, tumor location, involving thyroid isthmus, ultrasonic echo, unclear nodule border, irregular nodule morphology, microcalcification, tumor vascularity, multiple nodules, bilateral nodules, bilateral focality, multifocality, capsular invasion, capsular dorsal invasion, extrathyroidal extension, and Hashimoto’s thyroiditis.

Tumor size was defined as the maximum tumor diameter in unifocal cases, or the maximal diameter of the largest tumor in multifocal cases on ultrasound (12). Malignant lesions in the isthmus have been found to be associated with a higher rate of multifocality, capsular invasion, extra-thyroidal extension, and lymph node metastasis (13). Ultrasound feature of hypoechoic thyroid nodules is highly correlated with an increased risk of malignancy. Unclear nodule border referred to tumor nodules whose margins were not well defined under ultrasound. Irregular shape meant that the ratio of anterior to posterior diameter to horizontal diameter was greater than 1 when measured transversely. Microcalcifications were defined as dotted echogenic lesions ≤1 mm within the tumor. Tumor vascularity indicated an obvious blood flow signal in the tumor when using color Doppler flow imaging. Multiple nodules were identified when there were other nodules (benign or malignant) attached to the thyroid along with the primary tumor. In exceptional cases, when the additional nodules were situated in the opposite lobe of the primary tumor, they were referred to as bilateral nodules. If multiple nodules had suspicious malignant ultrasound features (scored higher than Thyroid Imaging Reporting and Data System (TI-RADS) 4A), we defined this condition as multifocality. And bilateral focality denoted suspicious malignancy involving both thyroid lobes. Also included were multiple features of the abnormal lateral lymph nodes on ultrasound, including lymph node size, shape, margins, echotexture, microcalcifications, and vascularity (14). All the above ultrasound characters were appraised by our sonographers who had over 10 years of experience in diagnosing thyroid ultrasound images.

### Machine learning techniques

Six well-established machine learning algorithms were used for modeling: the decision tree, the XGBoost, the random forest, the support vector machine, the neural network, and the K-nearest neighbor.

Decision tree, XGBoost, and random forest are tree-based nonlinear algorithms. The growth of the tree is carried out by repeated binary splits of the data. Starting with the data represented by a single node at the top of the tree, the splitting process is repeated (binary splitting), and the subnode is then further split into two child nodes, repeating the process until the “tree” is fully grown (achieving “node purity, i.e. all leaf nodes contain only samples from one class”). When running the output of results, they are usually combined by ‘voting’, i.e. each tree in the forest casts a vote for the classification of the new sample, with the winner being the category with the most votes. A support vector machine is a binary classifier that implicitly maps inputs to a high-dimensional feature space *via* a non-linear transformation (also known as the kernel trick), and applies a linear decision surface in the optimal hyperplane to discriminate between classes. According to Batta, a neural network is a functional network designed to identify potential relationships in a set of data, a process inspired by mimicking the way the human brain works. When analyzing data, the neural network studies from labeled examples (i.e. data with ‘answers’), and is capable of approximating arbitrary functions with arbitrary precision, achieving the full internet implications of the word ‘smart’. The K-Nearest Neighbor algorithm, proposed by Cover and Hart, is a non-parametric classification method. When used for classification, the k-nearest neighbor algorithm classifies a new observation into the majority class of its nearest neighbors.

Notably, we compared the predictive performance of these non-linear machine learning models with a traditional logistic regression-based Nomogram (15).

### Model development and evaluation

For efficient prediction, data were preprocessed as follows: A) The data were cleaned to identify any missing values (imputed by their arithmetic means), outliers, and duplicates. B) Feature selection was performed *via* LASSO regression to exclude potentially redundant covariates and reduce the impact of data overfitting. C) Continuous variables were normalized to zero mean and unit variance, while categorical variables were one-hot encoded. D) To mitigate data inequalities, we adopted the Synthetic Minority Oversampling Technique (SMOTE) algorithm, a commonly used algorithm that oversamples the minority, to balance the training set.

The whole dataset was randomly divided into a training set (80%) and a test set (20%). The training set was used for model construction by using different machine learning algorithms. During training, a GridSearch method with 5-fold cross-validation was applied for optimization to reduce prediction errors. In the test set, the evaluation parameters were measured as follows:

-
$\text{Precision}=\frac{\text{TP}}{\text{TP}+\text{FP}}$

-
$\text{Recall}=\frac{\text{TP}}{\text{TP}+\text{FN}}$

-
$\text{Accuracy}=\frac{\text{TP}+\text{TN}}{\text{TP}+\text{FP}+\text{TN}+\text{FN}}$

-
$\text{F}1\text{ score}=\frac{2*\text{Precision}*\text{Recall}}{\text{Precision}+\text{Recall}}$

-
$\text{Specificity}=\frac{\text{TN}}{\text{TN}+\text{FP}}$

-
$\text{Sensitivity}=\frac{\text{TP}}{\text{TP}+\text{FN}}$

TP, FP, TN, and FN mean true positive, false positive, true negative and false negative respectively.

In addition, we validated the performance of the machine learning models in the internal test set by applying the receiver operating characteristic (ROC) curve and decision curve analysis (DCA). The area under the curve (AUC) of ROC was measured to show the discriminatory power of the models, while the DCA assessed the net benefit in clinical utility.

### Interpretable machine learning

To further understand how each feature contributes to the classification, we introduced the SHAP package to interpret the output of the machine learning model through a game theoretic approach as a way to assess the feature importance in machine learning methods. To gain insight into the interaction of variables on classification, we used the ‘Seaborn’ library in Python (a Matplotlib-based Python data visualization library) to explore the effect of variables on model outputs. In addition, we developed a web-based tool for clinicians to use the compact model.

### Statistical analysis

The code for our machine learning is written using the following packages: Numpy, Pandas, Matplotlib, Scikit-learn, Seaborn, and SHAP packages, under the python programming language in version 3.8. Descriptive statistics were presented as means with (standard deviation), median (interquartile range), or number (percentage), and univariate analysis was performed with Student’s t test, Mann-Whitney U test, Pearson chi-square test, or Fisher exact test, as appropriate. Variables with a P value<0.05 in univariate analysis were included in the multivariate analysis (LR forward). Univariate and multivariate analysis was performed using IBM SPSS 25.0 (version 25.0; Armonk, NY, USA). Differences were considered statistically significant at P<0.05.

## Results

### Patients and disease characteristics

**Table 1** demonstrates the characteristics of the 1815 patients who underwent lateral lymph node dissection. Of these patients, 670 (36.915%) were male. The median (IQR) age was 42 (33-51) years; the median (IQR) body mass index was 24.47 (22.03-27.10). 270 (14.88%) had a smoking history, 376 (20.72%) were alcohol drinkers, and 65 (3.58%) had a family thyroid cancer history. There were 292 (16.09%) patients with hypertension and 115 (6.34%) with diabetes. The mean (SD) of Tg-Ab and TPO-Ab were 99.14 (221.24) and 221.63 (427.56) respectively. The mean (SD) of tumor size was 1.54 (0.99), 471 (35.33%) tumors were located in the upper pole, 184 (10.19%) invaded the isthmus, 1432 (78.90%) showed tumor microcalcification, 1264 (69.64%) had vascularity, 562 (30.96%) presented with multifocality, and 1511 (83.25%) were found to have abnormal lymph nodes by preoperative ultrasound. Additionally, the detailed clinical characteristics of training and test sets could be found in **Supplementary Table 1**.

**Table 1:** Univariate analysis of clinical characteristics related to LLMN.

Characteristic	Overall	LLMN (−)	LLMN (+)	p-Value
**Patient population, n**	**1815**	**680**	**1135**	
**Demographic data**				
Male, n (%)	670 (36.92)	222 (32.65)	448 (39.47)	0.004^*^
Age, median (IQR) (y)	42.00 (33.00,51.00)	45.00 (36.00,53.00)	40.00 (31.00,49.00)	<0.001^*^
Height, median (IQR) (m)	165.00 (160.00,172.00)	165.00 (160.00,170.00)	166.00 (160.00,172.00)	<0.001^*^
Weight, median (IQR) (kg)	67.00 (59.00,77.00)	66.60 (60.00,76.00)	67.00 (58.50,78.00)	0.711
BMI, median (IQR) (kg/m^2^)	24.47 (22.03,27.10)	24.54 (22.43,26.78)	24.34 (21.80,27.26)	0.235
Smoking, n (%)	270 (14.88)	73 (10.74)	197 (17.36)	<0.001^*^
Alcohol, n (%)	376 (20.72)	128 (18.82)	248 (21.85)	0.124
Menopause, n (%)	980 (53.99)	380 (55.88)	600 (52.86)	0.212
Hypertension, n (%)	292 (16.09)	130 (19.12)	162 (14.27)	0.007^*^
Diabetes mellitus, n (%)	115 (6.34)	51 (7.50)	64 (5.64)	0.115
Dyslipidemia, n (%)	14 (0.77)	5 (0.74)	9 (0.79)	0.892
Personal cancer history, n (%)	47 (2.59)	20 (2.94)	27 (2.38)	0.465
Family thyroid cancer history, n (%)	65 (3.58)	17 (2.50)	48 (4.23)	0.055
Family other cancer history, n (%)	144 (7.93)	68 (10.00)	76 (6.70)	0.012^*^
SBP, median (IQR) (mmHg)	119.00 (109.00,130.00)	121.00 (111.00,131.00)	117.00 (108.00,129.00)	<0.001^*^
DBP, median (IQR) (mmHg)	75.00 (68.00,82.00)	76.00 (69.00,83.00)	74.00 (67.00,82.00)	0.003^*^
MAP, median (IQR) (mmHg)	89.67 (82.00,98.00)	91.00 (83.33,99.00)	88.67 (81.00,97.33)	<0.001^*^
**Laboratory findings**				
HGB, mean (SD) (g/L)	135.05 (17.70)	134.71 (17.01)	135.25 (18.10)	0.523
WBC, mean (SD) (10^9^/L)	6.26 (1.84)	6.17 (1.68)	6.32 (1.92)	0.079
Neutrophil percentage, mean (SD) (%)	0.56 (0.08)	0.56 (0.08)	0.56 (0.08)	0.648
Lymphocyte percentage, mean (SD) (%)	0.35 (0.08)	0.35 (0.08)	0.35 (0.08)	0.352
NLR, mean (SD)	1.80 (1.28)	1.75 (0.82)	1.83 (1.49)	0.189
PLT, mean (SD) (10^9^/L)	241.68 (58.21)	237.71 (56.40)	244.07 (59.15)	0.025^*^
ALT, mean (SD) (U/L)	19.65 (15.35)	19.88 (15.24)	19.52 (15.42)	0.635
AST, mean (SD) (U/L)	16.72 (6.80)	17.18 (7.10)	16.44 (6.59)	0.025^*^
Albumin, mean (SD) (g/L)	42.95 (3.20)	42.76 (3.17)	43.06 (3.21)	0.055
Total protein, mean (SD) (g/L)	68.70 (4.91)	68.86 (4.86)	68.60 (4.94)	0.292
SCR, mean (SD) (umol/L)	68.97 (14.74)	68.41 (15.43)	69.30 (14.30)	0.215
BUN, mean (SD) (umol/L)	4.72 (1.47)	4.71 (1.21)	4.74 (1.60)	0.678
Serum potassium, mean (SD) (mmol/L)	3.99 (0.30)	3.98 (0.31)	3.99 (0.30)	0.285
Serum sodium, mean (SD) (mmol/L)	141.76 (2.23)	141.77 (2.27)	141.75 (2.21)	0.853
Blood glucose, mean (SD) (mmol/L)	4.91 (0.99)	4.97 (1.04)	4.88 (0.96)	0.069
TBIL, mean (SD) (umol/L)	11.49 (5.25)	11.40 (5.33)	11.54 (5.20)	0.585
DBIL, mean (SD) (umol/L)	3.24 (1.62)	3.15 (1.61)	3.30 (1.62)	0.072
LDL, mean (SD) (X)	2.69 (0.75)	2.74 (0.76)	2.67 (0.74)	0.109
HDL, mean (SD) (X)	1.19 (0.32)	1.18 (0.31)	1.20 (0.32)	0.420
APTT, mean (SD) (s)	36.29 (4.37)	36.04 (4.10)	36.44 (4.51)	0.059
PT, mean (SD) (s)	13.10 (1.06)	13.10 (1.35)	13.10 (0.85)	0.917
T3, mean (SD) (pg/ml)	1.65 (0.31)	1.66 (0.33)	1.64 (0.30)	0.110
T4, mean (SD) (pg/ml)	96.74 (19.26)	97.05 (19.32)	96.55 (19.22)	0.597
FT3, mean (SD) (pg/ml)	4.77 (0.63)	4.76 (0.62)	4.78 (0.64)	0.535
FT4, mean (SD) (pg/ml)	15.15 (2.36)	15.12 (2.29)	15.17 (2.40)	0.663
TSH, mean (SD) μIU/ml	2.73 (4.27)	2.66 (2.64)	2.76 (5.00)	0.640
Tg-Ab, mean (SD) IU/ml	99.14 (221.24)	85.53 (149.58)	107.47 (254.99)	0.026^*^
TPO-Ab, mean (SD) IU/ml	221.63 (427.56)	225.43 (429.02)	219.30 (426.65)	0.775
**Ultrasonography detail**				
Tumor size, mean (SD) (cm)	1.54 (0.99)	1.16 (0.68)	1.77 (1.07)	<0.001^*^
Tumor location, n (%)				
pUpper	471 (35.33)	135 (24.95)	336 (42.42)	<0.001^*^
Middle	498 (37.36)	230 (42.51)	268 (33.84)	
Lower	350 (26.26)	174 (32.16)	176 (22.22)	
Diffuse	14 (1.05)	2 (0.37)	12 (1.52)	
Involving thyroid isthmus, n (%)	184 (10.19)	83 (12.24)	101 (8.96)	0.026^*^
Ultrasonic echo, n (%)				
Hypoechoic	1730 (95.85)	660 (97.35)	1070 (94.94)	0.045^*^
Isoechoic	68 (3.77)	16 (2.36)	52 (4.61)	
Hyperechoic	7 (0.39)	2 (0.30)	5 (0.44)	
Unclear nodule border, n (%)	1274 (71.49)	478 (71.24)	796 (71.65)	0.853
Irregular nodule morphology, n (%)	1406 (81.60)	517 (80.53)	889 (82.24)	0.376
Microcalcification, n (%)	1432 (78.90)	481 (70.74)	951 (83.79)	<0.001^*^
Tumor vascularity, n (%)	1264 (69.64)	451 (66.32)	813 (71.63)	0.017^*^
Multiple nodules, n (%)	1311 (72.23)	494 (72.65)	817 (71.98)	0.760
Bilateral nodules, n (%)	1055 (58.13)	407 (59.85)	648 (57.09)	0.249
Bilateral focality, n (%)	398 (21.93)	134 (19.71)	264 (23.26)	0.077
Multifocality, n (%)	562 (30.96)	183 (26.91)	379 (33.39)	0.004^*^
Capsular invasion, n (%)				
Negative	1557 (85.79)	584 (85.88)	973 (85.73)	0.085
Proximity	101 (5.57)	46 (6.77)	55 (4.85)	
Invasion	157 (8.65)	50 (7.35)	107 (9.43)	
Capsular dorsal invasion, n (%)	76 (4.19)	19 (2.80)	57 (5.02)	0.022^*^
Extrathyroidal extension, n (%)	84 (4.63)	30 (4.41)	54 (4.76)	0.734
Hashimoto’s thyroiditis, n (%)	282 (15.54)	116 (17.06)	166 (14.63)	0.166
Abnormal LNs, n (%)	1511 (83.25)	502 (73.82)	1009 (88.90)	<0.001^*^
LNs ultrasonic echo, n (%)				
Hypoechoic	1491 (99.40)	496 (99.60)	995 (99.30)	0.483
Isoechoic	9 (0.60)	2 (0.40)	7 (0.70)	
LNs size, mean (SD) (cm)	1.54 (0.83)	1.29 (0.71)	1.67 (0.86)	<0.001^*^
Unclear LNs border, n (%)	90 (6.16)	22 (4.51)	68 (7.00)	0.062
Irregular LNs morphology, n (%)	306 (23.36)	72 (16.18)	234 (27.05)	<0.001^*^
Abnormal lymphatic portal structure, n (%)	209 (16.34)	101 (23.06)	108 (12.84)	<0.001^*^
LNs microcalcification, n (%)	629 (41.63)	135 (26.89)	494 (48.96)	<0.001^*^
LNs vascularity, n (%)	912 (60.36)	228 (45.42)	684 (67.79)	<0.001^*^

^*^ means p-value< 0.05. ALT, alanine aminotransferase; AST, aspartate aminotransferase; APTT, activated partial thrombin time; BMI, body mass index; BUN, blood urea nitrogen; DBIL, direct bilirubin; DBP, diastolic blood pressure; FT3, free T3; FT4, free T4; HDL, high density lipoprotein; HGB, hemoglobin; LDL, low density lipoprotein; LLNM, lateral lymph node metastases; LNs, lymph nodes; MAP, mean arterial pressure; NLR, neutrophil-to-lymphocyte ratio; PLT, platelet; PT, prothrombin time; SBP, systolic blood pressure; SCR, serum creatinine; T3, triiodothyronine; T4, tetraiodothyronine; TBIL, total bilirubin; Tg-Ab, thyroglobulin antibody; TPO-Ab, thyroid peroxidase antibody; TSH, thyroid stimulating hormone; WBC, white blood cell count.

Of the 1815 patients enrolled in this study, a total of 1135 (62.53%) experienced LLNM. To investigate the effect of risk factors on LLNM, we first investigated the relationship between clinical characteristics and LLNM by univariate analysis. In the logistic regression analysis, the following demographic data were significantly associated with LLNM: male, age, height, smoking, hypertension, family history of other cancers (excluding thyroid cancer), and mean arterial pressure. Among the ultrasound features, tumor size, tumor location, invasion of the isthmus, echotexture, microcalcifications, vascularity, and multifocality were significantly different between the patients with and those without LLNM (all P values< 0.05, **Table 1**). However, unclear nodule border, irregular nodule morphology, multiple nodules, bilateral nodules, and bilateral focality were not associated with LLNM. Notably, the number of capsular invasion was similar in patients with and without LLNM (P > 0.05), but capsular dorsal invasion showed statistically significant differences (P< 0.05). Extrathyroidal extension and Hashimoto’s thyroiditis correlated negatively with the risk of LLNM (P > 0.05). In addition, we found that abnormal lymph nodes detected preoperatively by ultrasound were positively associated with the risk of LLNM (P< 0.05).

Then, variables with P value<0.05 in univariate analysis were screened out for multivariate analysis using LR forward stepwise selection. The results showed that age (OR=0.96, 95% CI: 0.95-0.98, P<0.001), hypertension (OR=0.59, 95% CI: 0.36-0.97, P=0.039), smoking (OR=1.78, 95% CI: 1.09-2.92, P=0.022), tumor size (OR=2.63, 95% CI=1.97-3.52, P<0.001), tumor location (vs. Upper; middle, OR=0.40, 95% CI=0.27-0.60, P<0.001; lower OR=0.31, 95% CI=0.20-0.48, P<0.001), tumor vascularity (OR=0.63, 95% CI=0.43-0.91, P=0.015), capsular dorsal invasion (OR=2.90, 95% CI=1.23-6.81, P=0.015), lymph nodule size (OR=1.45, 95% CI=1.11-1.88, P=0.006), irregular lymph nodule morphology (OR=1.75, 95% CI=1.14-2.68, P=0.01), abnormal lymphatic portal structure (OR=0.56, 95% CI=0.36-0.85, P=0.007), lymph nodule microcalcification (OR=1.87, 95% CI=1.32-2.66, P<0.001), and lymph nodule vascularity (OR=2.20, 95% CI=1.55-3.13, P<0.001) showed significant correlations with LLNM in PTC patients (**Table 2**).

**Table 2:** Multivariate analysis of clinical characteristics related to LLMN.

Variable	OR	95% CI	p-Value
Age	0.96	0.95-0.98	<0.001^*^
Hypertension			
Yes	0.59	0.36-0.97	0.039^*^
No	Reference	–	–
Smoking			
Yes	1.78	1.09-2.92	0.022^*^
No	Reference	–	–
Tumor size	2.63	1.97-3.52	<0.001^*^
Tumor location			
Upper	Reference	–	–
Middle	0.40	0.27-0.60	<0.001^*^
Lower	0.31	0.20-0.48	<0.001^*^
Tumor vascularity			
Yes	0.63	0.43-0.91	0.015^*^
No	Reference	–	–
Capsular dorsal invasion			
Yes	2.90	1.23-6.81	0.015^*^
No	Reference	–	–
LNs size	1.45	1.11-1.88	0.006^*^
Irregular LNs morphology			
Yes	1.75	1.14-2.68	0.01^*^
No	Reference	–	–
Abnormal lymphatic portal structure			
Yes	0.56	0.36-0.85	0.007^*^
No	Reference	–	–
LNs microcalcification			
Yes	1.87	1.32-2.66	<0.001^*^
No	Reference	–	–
LNs vascularity			
Yes	2.20	1.55-3.13	<0.001^*^
No	Reference	–	–

^*^ means p-value< 0.05. LNs, lymph nodes.

### Feature selection

**Figure 3:**
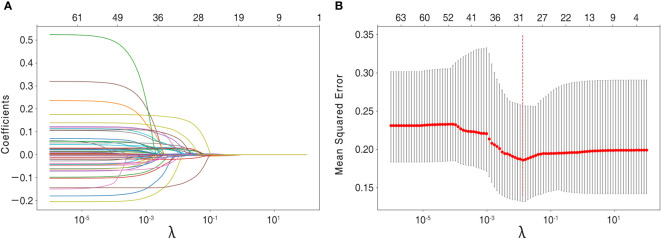
Feature selection using the LASSO regression model. **(A)** Lasso regression analysis coefficients. **(B)** For feature selection, the penalty parameter λ was chosen using the LASSO method, with the minimal mean squared error as the criterion. Dotted vertical lines were drawn on the optimal values and a value of λ of 0.013was chosen, with the optimal λ leading to 30 non-zero coefficients in this study.

The recruited 69 features summarized in **Table 1** were subjected to feature selection by the LASSO regression, using mean squared error and minimum λ as the criteria. In **Figure 3**, when the mean squared error was minimal (λ = 0.013 at this point), there were 30 non-zero features in the LASSO regression, which were identified as follows: Continuous variables include alanine aminotransferase, aspartate aminotransferase, age, albumin, body mass index, blood urea nitrogen, blood glucose, direct bilirubin, hemoglobin, height, low-density lipoprotein, lymph nodule size, neutrophil-to-lymphocyte ratio, serum potassium, serum sodium, systolic blood pressure, T3, T4, total bilirubin, Tg-Ab, total protein, tumor size, and white blood cell count; Categorical variables include capsular invasion, irregular lymph nodule morphology, lymph nodule microcalcification, lymph nodule vascularity, tumor microcalcification, multifocality, and tumor location.

These 30 features with non-zero weighting factors were admitted and used to develop the final machine learning models.

### Model performance and clinical practicality

**Figure 4:**
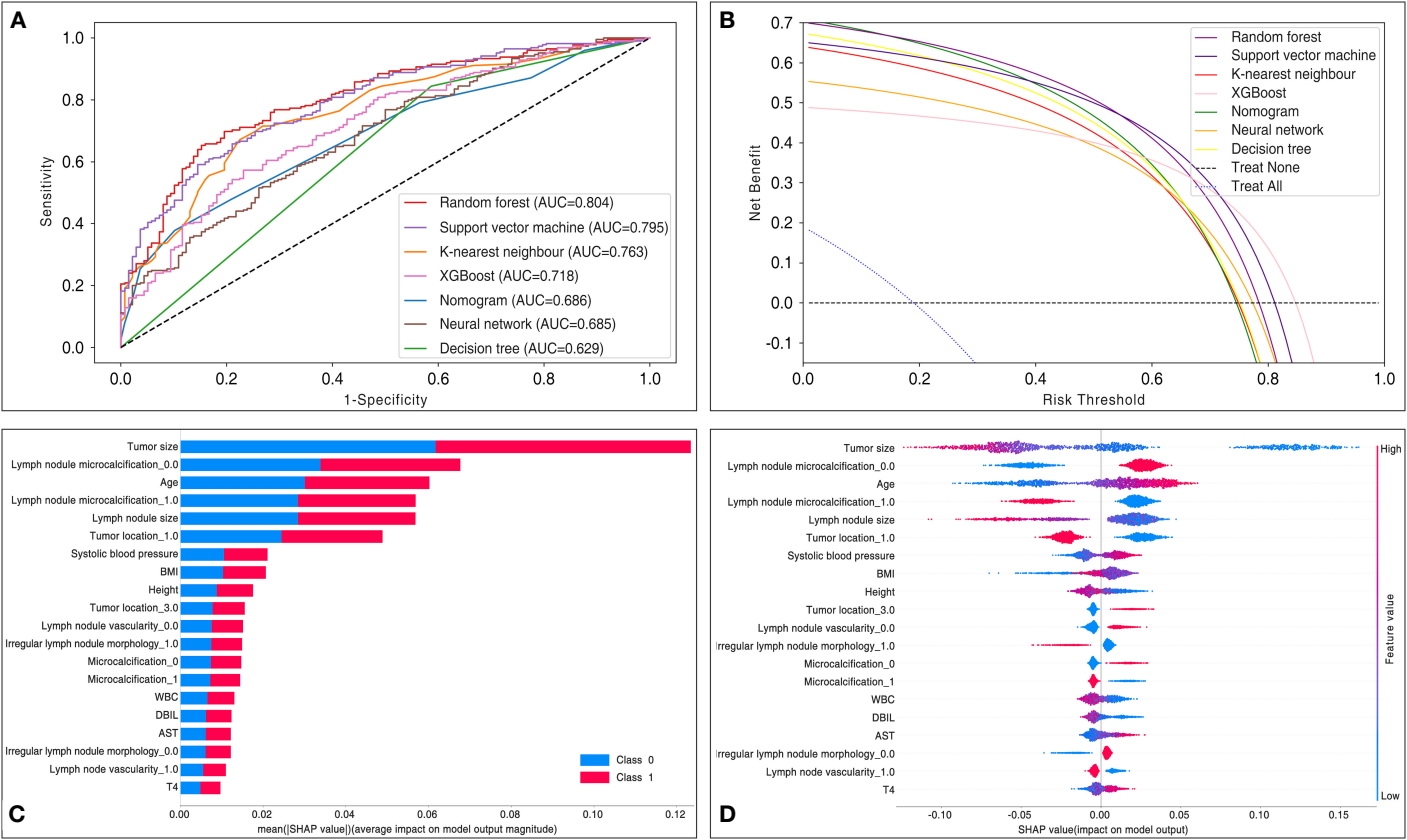
Comparisons in model performance between six machine learning and traditional logistic regression-based Nomogram. **(A)** Receiver operating characteristic curve display a comparison of the predictive model discrimination based on AUC scores. **(B)** Decision curve analysis assessed the net benefit of the models in terms of clinical utility. The decision curve analysis mapped the net benefit (y-axis) versus the risk threshold (x-axis). It mimicked two scenarios: the black dashed line represented the expected net benefit relative to ‘no intervention’, while the blue dashed line represented the expected net benefit relative to ‘full intervention’. The decision curve analysis indicated that each predictive model had a higher net benefit than the ‘all treatment’ or ‘no treatment’ strategies under different probability thresholds. AUC = area under the curve. **(C)** The SHAP evaluated a given feature by assessing its contribution to the prediction. The average contribution of the top 20 variables to the magnitude of the model output was ordered according to the descending order of their average absolute contribution to the classification. **(D)** Each point represents the SHAP value for a particular feature of a particular patient. The further a point is from the x-axis (positive or negative x), the greater the impact of this attribute on the output. The color represents the high (red) and low (blue) original feature values, as indicated by the color array stripes on the right. AST, aspartate aminotransferase; AUC, area under the curve; BMI, body mass index; DBIL, direct bilirubin; T4, tetraiodothyronine; WBC, white blood cell count.

**Table 3** and **Figure 4A** summarize the performance of different models in predicting mortality, including the traditional logistic regression-based Nomogram and different machine learning models, accompanied by AUC scores fluctuating from 0.63 to 0.80. Of all the models tested, the random forest had the most robust predictive ability with regard to AUC (0.80), coupled with better accuracy (0.74), precision (0.74), recall (0.89), F1 score (0.81), and sensitivity (0.89), although the specificity was only 0.49. The AUC metrics for the support vector machine, K-nearest neighbor, XGBoost, neural network, and decision tree were 0.80, 0.76, 0.72, 0.69, and 0.63, respectively. Of note, the traditional logistic regression-based Nomogram yielded an AUC metric of 0.69, and the AUC difference between it and the random forest was statistically significant (tested by the DeLong test).

**Table 3:** The performance for each of the models.

	**Decision tree**	**Random forest**	**XGboost**	**Support vector machine**	**Neural network**	**K-nearest neighbors**	**Nomogram**
Accuracy	0.68	0.74	0.63	0.73	0.66	0.70	0.66
Precision	0.70	0.74	0.79	0.77	0.70	0.70	0.70
Recall	0.84	0.89	0.55	0.81	0.79	0.90	0.79
F1 score	0.77	0.81	0.65	0.79	0.74	0.79	0.74
Sensitivity	0.84	0.89	0.55	0.81	0.79	0.90	0.79
Specificity	0.41	0.49	0.77	0.60	0.44	0.36	0.44
AUC score	0.63	0.80*	0.72	0.80	0.69	0.76	0.69

*Statistical significance of differences in AUC scores between Random forest and Nomogram (tested by the DeLong test). AUC, area under the curve.

To determine the clinical benefit of the models, we generated DCA. The DCA maps the net benefit (y-axis) versus the risk threshold (x-axis). It mimics two scenarios: the black dashed line represents the expected net benefit relative to ‘no intervention’, while the blue dashed line represents the expected net benefit relative to ‘full intervention’. As the threshold probability may differ from patient to patient, the net benefit is calculated over a range of probabilities. Results from the DCA show that all models, including Nomogram, hold higher net clinical benefit than the two extreme lines in the reasonable threshold range of 0 to 0.8 (**Figure 4B**). Specifically, random forest yielded a consistently high net benefit within a reasonable range of threshold probability.

### Data visualization

We performed interpretable machine learning by using the SHAP method in the best model, the random forest. The importance ranking of the most influential features on the model output is shown in **Figures 4C, D**. By using the SHAP approach, we identified the features that contributed the most to the model runs, where the top 5 variables were ranked as follows: tumor size, lymph node microcalcification, age, lymph node size, and tumor location. Interestingly, in contrast to the variables in **Table 2**, we found that all the top 5 variables were also identified as the most relevant risk factors in the multivariate analysis.

**Figure 5:**
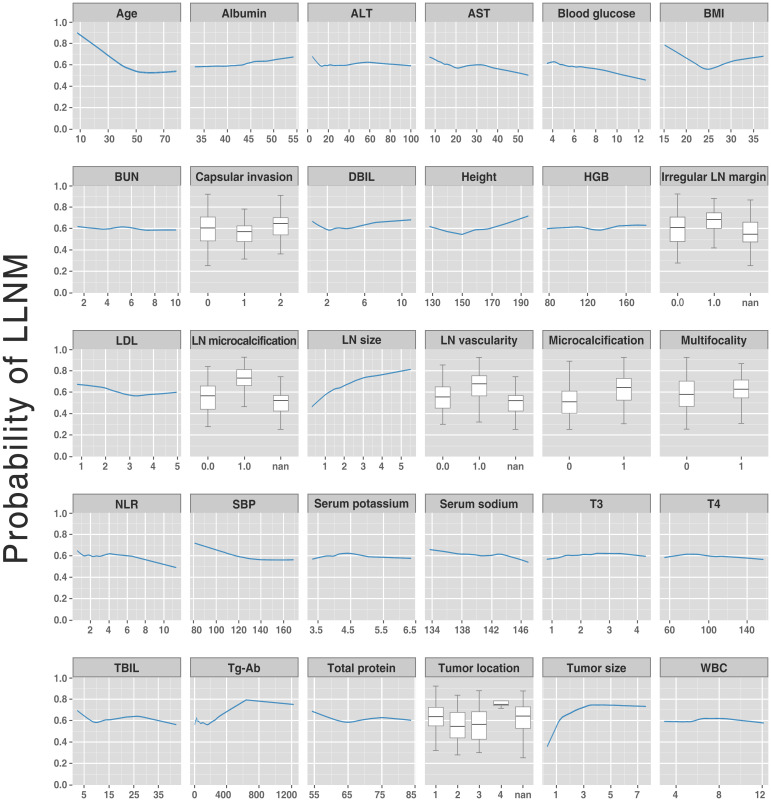
Partial dependent plot (for continuous variables) and box plots (for categorical variables) showing LLNM probabilities vs. variable values for the 30 variables. The y-axis denotes the predicted LLNM probability (range: 0 to 1). The x-axis spans the range (or category) of the 30 predictors. LLNM, lateral lymph node metastases.

Variable dependence plots were generated to better understand how the original values of 30 variables affect the model output (**Figure 5**). The generated plots consist of curves (for continuous variables) and box plots (for categorical variables) of LLNM probabilities versus variable values for the 30 predictors, showing changes in variable contributions as their values span the range in the plot. Most fascinatingly, regarding the risk probability of LLNM, we observed that the calculated optimal cut-off threshold for tumor size and age were 1 cm and 55 years, respectively.

### Website-based tool

**Figure 6:**
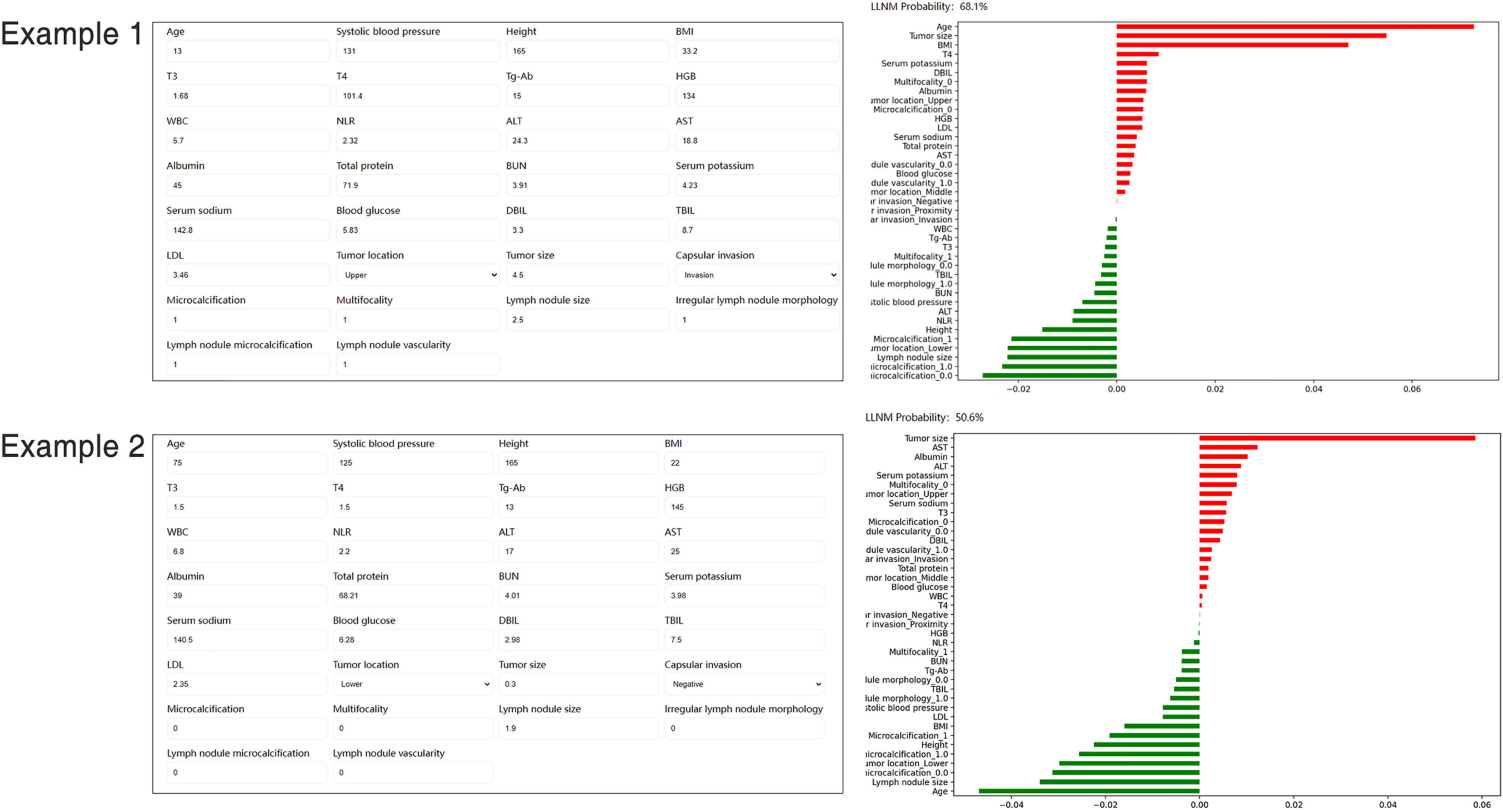
Screenshot of examples from the website tool. Input values for key variables to determine the risk of LLNM and show the contribution of each value for the model output. LLNM, lateral lymph node metastases.

A website was established for clinicians to use the proposed model, http://43.138.62.202/. By using this tool the LLNM can be evaluated, and the interpretation of the results at an individual level can also be visualized to the users. Two examples of individuals who were correctly predicted to develop LLNM or not were shown in **Figure 6**.

## Discussion

The incidence of PTC is currently showing rapid growth worldwide. Although the prognosis of patients with PTC is excellent, with a 10-year survival rate of more than 90% (16), early LLNM is quite common. Between 20% and 69% of patients with stage N0 PTC have been reported to have subclinical LLNM (17). In the present study, 1135 (62.53%) of the 1815 patients included underwent LLNM. Previous studies have shown that LLNM correlates significantly with local recurrence and survival (18). However, there is still much debate about the propriety of prophylactic resection of negative lateral lymph nodes. Proponents of this view hold that metastasis to LLNM is associated with poor prognosis and recurrence, while opponents argue that prophylactic surgery increases the risk of complications such as nerve injury, chyle leak, shoulder ache, limited mobility, and hypoparathyroidism (19). Chung et al. concluded that the incidence of surgery-related complications was significantly higher in patients who underwent lateral lymph node dissection than in those who did not (20). According to the latest ATA guidelines from 2015 (9), prophylactic lateral lymph node resection is not recommended for patients with PTC without evidence of LLNM on clinical examination or imaging. Tragically, ultrasound/computed tomography is highly specific but of low sensitivity in identifying LLNM (7, 21), and a 30% false-negative-rate for benign fine-needle-aspiration-biopsy results has been reported (22). Moreover, occult LLNM may occur and not be detected by preoperative regular examination (23). Therefore, clinicians call for accurate and objective tools to ascertain whether LLNM has occurred.

No reliable predictive model for LLNM in PTC patients currently exists, and the accurate diagnosis of LLNM relies heavily on postoperative pathology. Screening for those at high risk of LLNM is necessary for discriminating patients who may require prophylactic lateral lymph node dissection. Traditional nomograms incorporate multiple independent variables to create models enabling prediction of the clinical events, thus aiding clinicians in decision analysis. Many previous studies exploring risk factors for LLNM in PTC have proposed several nomograms to quantitatively assess the probability of metastasis. For example, Jin et al. (24) and Wang et al. (18) developed nomograms based on clinicopathological factors. Unfortunately, these nomograms are not available for preoperative assessment as they are mostly based on postoperative pathological features and therefore, cannot be used in a preoperative context. Recently, Zhuo et al. proposed a nomogram integrating six identified preoperative risk factors: sex, tumor size, multiple nodules, tumor shape, lymph node vascularity, and lymph node location (15). In this study, we validated this nomogram and presented its performance through various parameters (e.g., ROC and DCA for predictive performance and clinical utility, respectively), accompanied by further comparisons of the performance with multiple machine learning models.

A retrospective cohort of 1815 patients with PTC comprised the whole dataset. Previous studies reported that feature optimization enabled the predictive value of features to be improved. Our study enrolled multiple lesion- and lymph node-relevant features, including size, location, multifocality, local infiltration intensity-related features, shape-based profiles, and echotexture features, all of which should represent the underlying tumor biology. LASSO is a regression analysis method that simultaneously performs feature selection and regularization to improve prediction accuracy (25). LASSO has proven to be a promising optimization feature selection method (26). Therefore, we screened out 30 candidate variables by LASSO regression to construct the model and developed web tool based on them. The result of our study suggests that random forest yielded a higher AUC than that of other machine learning algorithms. This finding concurs with the literature. The random forest has several advantages over other machine learning algorithms: its resistance to overfitting, its accommodations for both continuous and categorical variables, its allowance for estimating error rates, and its capability to rank variables by relative importance. In addition, this tree-based ensemble algorithm offers the widest coverage for various classification tasks (27). The innovation of this study is that by integrating machine learning algorithms, several predictive LLNM models were developed and further compared with the traditional logistic regression-based nomogram. The predictive performance of the random forest outperformed the nomogram, indicating that random forest is the optimal and novel model for predicting LLNM. A possible reason is that the machine learning algorithm analyses other potential non-linear associations about LLNM, which are ignored by traditional logistic regression.

Apart from generating a new machine learning model, we also explored the correlation between several risk factors and LLNM. Despite the fact that the correlation between variables and outcomes is invisible in most machine learning-based models, the ranked importance of variables in the optimal model was obtained by using a classifier-specific explainer (**Figures 4C, D**). Of these, the top 5 variables were considered to be the most important risk factors for LLNM, with them being: tumor size, lymph node microcalcifications, age, lymph node size, and tumor location. In previous studies, factors associated with LLNM in PTC patients included age, gender, tumor size, tumor location, multiplicity, lymph node location, lymph node vascularity, capsular invasion, and extrathyroidal extension (28). It is well established that tumor size is associated with LLNM. Tumor size is usually positively correlated with the risk of LLNM, and the metastatic rate rises as the tumor diameter increases. However, all studies are at odds in defining the cut-off tumor size. Feng et al. (29) and Zhou et al. (15) considered tumors >1.0 cm as a risk factor for LLNM (16, 17), Wu et al. suggested a tumor size threshold of >0.7 cm (30), while Kim et al. (31) reported that PTC >2 cm was a strong independent risk factor for LLNM. Tumor size on ultrasound images is an important indicator of tumor growth. In the present study, tumor size ranked first in the mean ranking of machine learning model. In **Figure 5**, the original values of the tumor size vs. the risk of LLNM suggested that the optimal cut-off tumor size could be 1 cm. However, more clinical studies are needed to find the optimal cut-off threshold. Furthermore, age is commonly used to evaluate the grades of differentiated thyroid cancer. As noted by Lu et al, younger patients with PTC are more prone to develop LLNM than older patients (32). Such conditions may be associated with reduced tumor activity and the presence of occult metastases. However, although various staging systems list age as a predictor of PTC prognosis, the optimal cut-off in LLNM is still controversial. In a previous meta-analysis, patients with PTC under 45 years of age were found to be associated with an increased risk of LLNM (23). Traditionally, age<45 years has been a widely used clinical marker of prognosis in patients with PTC. However, in the 8th AJCC staging system, age<55 years was proposed as a more suitable prognostic cut-off than age<45 years (9). The present study showed that age was ranked third in the model, with a calculated optimal cut-off age of 55 years, which is identical to the 8th AJCC staging system. It is reasonable that younger patients with PTC should receive more attention. However, larger prospective studies are needed to clarify the exact relationship between age and tumor progression. Our study also showed that the risk of LLNM was significantly increased when the tumor location was in the upper pole of the thyroid. It has been reported that tumor location in the upper third of the thyroid is more likely to metastasize to grade II or III nodules on the side of the neck (23). The explanation may be that tumors located at the upper pole migrate through the lymphatics surrounding the superior thyroid artery (33). Rapidly proliferating malignancy is often accompanied by the occurrence of microcalcification. The present study found that lymph node size and microcalcification may be potential risk factors for LLNM. Therefore, PTC patients with these ultrasound features should be more carefully evaluated before surgery.

One of the main challenges of machine learning is the difficulty in understanding the rationale underlying the obtained results, thus limiting their utility for clinicians. Nowadays, web-based calculators offer greater convenience. This study presents the first online, freely accessible web server based on the proposed model to, at the individual level, quantify the risk of LLNM in PTC. By using our web-based tool, clinicians could obtain personalized information about the likelihood of LLNM before surgery. Identifying clinical risk factors for LLNM provides evidence for clinicians to optimize treatment strategies, e.g., to protect low-risk patients with stage N0/N1a PTC from complications caused by excessive surgery, such as chylous fistula or vagus nerves injury; or to offer one-time-therapeutic-resection-approach for PTC patients with high-risk LLNM at the time of initial surgery, thereby mitigating the need for additional surgery and meliorating prognostic outcomes.

Apart from the clinical implication, several methodological innovations were introduced in the present study. Firstly, it is the first study that develops machine learning-based models to predict LLNM in patients with PTC. In addition to routinely available clinical data, our study enrolled multiple lesion- and lymph node-relevant features on ultrasound, all of which may help reveal the underlying tumor biology. Based on these features, our models yielded favorable prediction performance and clinical utility as indicated by ROC curves and DCA. Then, using interpretable algorithms, we observed the variables ranking in LLNM to indicate their importance to model output. Next, to support clinicians in better understanding this novel model, we used partial dependency plots to interpret the model and visualize trends in LLNM risk. In the future, the online application of the developed compact model allows clinicians and patients in other hospitals to benefit from the present study.

However, this study does subject to several limitations. Firstly, its retrospective and monocentric design may limit generalizability. The observed model performance might vary across larger dataset with different distribution of sample features given that the samples used were only obtained from one medical centers. Secondly, despite the fact that feature selection reduces the over-fitting error and the impact of noise and random error, some potentially important variables may have been omitted during this process. In addition, although we demonstrated the potential feasibility of applying machine learning-guided risk stratification of LLNM in PTC patients, the study was further limited by the lack of external validation. It is not clear whether the results can be translated to generation of clinical benefits for patients, necessitating further prospective explorations.

## Conclusions

With the development of machine learning technology, it offers new ideas for clinical diagnosis and treatment of PTC. Incorporating machine learning methods into clinical routines can aid the clinician in decision-making and provide a “second opinion”, which may improve patient prognosis. This study is the first attempt employing machine learning models to predict LLNM in PTC patients. Notably, a web-based server has been developed to further improve its utility to clinicians, but its clinical implications and applications need further clarification. We believe that on the basis of this study, better algorithms will be available for clinical disease prediction.

## Data availability statement

The raw data supporting the conclusions of this article will be made available by the authors, without undue reservation.

## Ethics statement

The studies involving human participants were reviewed and approved by the Ethics Committee of the Chinese PLA General Hospital (S2022-431-01). Informed consent was waived due to the observational nature of the study.

## Author contributions

Conceptualization: S-WL, Y-LF, Y-HZ, and FZ. Methodology: ZW. Software: ZG. Validation: BW. Formal analysis: Y-LF. Investigation: Y-LF and S-WL. Resources: FZ and ZG. Data curation: P-LL, NY, and H-DQ. Writing—original draft preparation: S-WL, Y-LF, Y-HZ, and FZ. Writing—review and editing: P-LL, NY, and H-DQ. Visualization: ZG. Supervision: ZW. Project administration: H-DQ. All authors contributed to the article and approved the submitted version.

## Funding

This research received no external funding.

## Acknowledgments

The authors would like to acknowledge Mrs. Yuanyuan Cai for her help in coding and machine learning interpretation.

## Conflict of interest

The authors declare that the research was conducted in the absence of any commercial or financial relationships that could be construed as a potential conflict of interest.

## Publisher’s note

All claims expressed in this article are solely those of the authors and do not necessarily represent those of their affiliated organizations, or those of the publisher, the editors and the reviewers. Any product that may be evaluated in this article, or claim that may be made by its manufacturer, is not guaranteed or endorsed by the publisher.
