# Supplementary material for: Machine learning-based dynamic prediction of lateral lymph node metastasis in patients with papillary thyroid cancer
Source: Front Endocrinol (Lausanne). 2022 Oct 10;13:1019037. doi: 10.3389/fendo.2022.1019037 (PMC9589512; doi:10.3389/fendo.2022.1019037)
Supplement: Supplementary file 1 [file Table_1.docx]

**Supplemental Table 1. Comparison of clinical characteristics related to training set and test set.**

| **Characteristic** |  | **Training set** | **Test set** | **p-Value** |
| --- | --- | --- | --- | --- |
| **Patient population, n** |  | **1452** | **363** |  |
| **Demographic data** |  |  |  |  |
| Male, n (%) |  | 542(37.30) | 128(35.36) | 0.493 |
| Age, median (IQR) (y) |  | 42.00(33.00,51.00) | 41.00(32.00,51.00) | 0.412 |
| Height, median (IQR) (m) |  | 165.00(160.00,172.00) | 165.00(160.00,172000) | 0.383 |
| Weight, median (IQR) (kg) |  | 67.00(59.00,77.80) | 65.00(58.00,75.70) | 0.167 |
| BMI, median (IQR) (kg/m^2^) |  | 24.49(22.04,27.18) | 24.34(21.91,26.78) | 0.334 |
| Smoking, n (%) |  | 219(15.07) | 51(14.09) | 0.638 |
| Alcohol, n (%) |  | 302(20.79) | 74(20.44) | 0.886 |
| Menopause, n (%) |  | 796(54.78) | 184(50.83) | 0.177 |
| Hypertension, n (%) |  | 238(16.38) | 54(14.92) | 0.498 |
| Diabetes mellitus, n (%) |  | 97(6.68) | 18(4.97) | 0.234 |
| Dyslipidemia, n (%) |  | 11(0.76) | 3(0.83) | 0.889 |
| Personal cancer history, n (%) |  | 40(2.75) | 7(1.93) | 0.38 |
| Family thyroid cancer history, n (%) |  | 46(3.17) | 19(5.25) | 0.056 |
| Family other cancer history, n (%) |  | 114(7.85) | 30(8.29) | 0.781 |
| SBP, median (IQR) (mmHg) |  | 119.00(109.00,130.00) | 117.00(108.00,127.00) | 0.029^*^ |
| DBP, median (IQR) (mmHg) |  | 75.00(68.00,83.00) | 74.00(67.00,81.00) | 0.126 |
| MAP, median (IQR) (mmHg) |  | 89.67(82.33,98.33) | 88.67(80.67,96.67) | 0.047^*^ |
| **Laboratory findings** |  |  |  |  |
| HGB, mean (SD) (g/L) |  | 135.30(17.87) | 134.05(16.99) | 0.233 |
| WBC, mean (SD) (10^9^/L) |  | 6.26(1.91) | 6.26(1.52) | 0.967 |
| Neutrophil percentage, mean (SD) (%) |  | 0.56(0.08) | 0.56(0.08) | 0.58 |
| Lymphocyte percentage, mean (SD) (%) |  | 0.35(0.08) | 0.35(0.08) | 0.898 |
| NLR, mean (SD) |  | 1.81(1.36) | 1.77(0.85) | 0.598 |
| PLT, mean (SD) (10^9^/L) |  | 241.62(57.58) | 241.92(60.66) | 0.931 |
| ALT, mean (SD) (U/L) |  | 19.93(15.69) | 18.56(13.84) | 0.13 |
| AST, mean (SD) (U/L) |  | 16.80(6.96) | 16.40(6.08) | 0.318 |
| Albumin, mean (SD) (g/L) |  | 42.99(3.25) | 42.76(2.99) | 0.206 |
| Total protein, mean (SD) (g/L) |  | 68.79(4.96) | 68.34(4.71) | 0.12 |
| SCR, mean (SD) (umol/L) |  | 69.10(14.90) | 68.45(14.06) | 0.458 |
| BUN, mean (SD) (umol/L) |  | 4.68(1.26) | 4.81(1.18) | 0.071 |
| Serum potassium, mean (SD) (mmol/L) |  | 3.99(0.31) | 3.95(0.27) | 0.02^*^ |
| Serum sodium, mean (SD) (mmol/L) |  | 141.80(2.24) | 141.61(2.7) | 0.137 |
| Blood glucose, mean (SD) (mmol/L) |  | 4.92(1.01) | 4.88(0.89) | 0.445 |
| TBIL, mean (SD) (umol/L) |  | 11.48(5.16) | 11.52(5.58) | 0.894 |
| DBIL, mean (SD) (umol/L) |  | 3.22(1.61) | 3.35(1.64) | 0.172 |
| LDL, mean (SD) (X) |  | 2.71(0.76) | 2.65(0.71) | 0.249 |
| HDL, mean (SD) (X) |  | 1.19(0.31) | 1.19(0.33) | 0.909 |
| APTT, mean (SD) (s) |  | 36.22(4.26) | 36.57(4.75) | 0.17 |
| PT, mean (SD) (s) |  | 13.08(0.80) | 13.18(1.77) | 0.308 |
| T3, mean (SD) (pg/ml) |  | 1.64(0.30) | 1.65(0.35) | 0.874 |
| T4, mean (SD) (pg/ml) |  | 96.43(19.20) | 97.97(19.44) | 0.182 |
| FT3, mean (SD) (pg/ml) |  | 4.76(0.62) | 4.80(0.67) | 0.314 |
| FT4, mean (SD) (pg/ml) |  | 15.18(2.40) | 15.02(2.17) | 0.267 |
| TSH, mean (SD) μIU/ml |  | 2.77(4.66) | 2.55(2.05) | 0.381 |
| Tg-Ab, mean (SD) IU/ml |  | 99.95(235.05) | 95.93(154.39) | 0.765 |
| TPO-Ab, mean (SD) IU/ml |  | 215.53(421.79) | 245.97(449.03) | 0.241 |
| **Ultrasonography detail** |  |  |  |  |
| Tumor size, mean (SD) (cm) |  | 1.54(0.98) | 1.55(1.00) | 0.858 |
| Tumor location, n (%) |  |  |  |  |
| Upper |  | 379(35.55) | 92(34.46) | 0.642 |
| Middle |  | 390(36.59) | 108(40.45) |  |
| Lower |  | 285(26.74) | 65(24.35) |  |
| Diffuse |  | 12(1.13) | 2(0.75) |  |
| Involving thyroid isthmus, n (%) |  | 153(10.60) | 31(8.59) | 0.259 |
| Ultrasonic echo, n (%) |  |  |  |  |
| Hypoechoic |  | 1385(95.91) | 1385(95.91) | 0.844 |
| Isoechoic |  | 54(3.74) | 14(3.88) |  |
| Hyperechoic |  | 5(0.35) | 2(0.55) |  |
| Unclear nodule border, n (%) |  | 1019(71.36) | 255(72.03) | 0.801 |
| Irregular nodule morphology, n (%) |  | 1133(81.75) | 273(81.01) | 0.754 |
| Microcalcification, n (%) |  | 1157(79.63) | 275(75.97) | 0.127 |
| Tumor vascularity, n (%) |  | 1013(69.72) | 251(69.34) | 0.888 |
| Multiple nodules, n (%) |  | 1051(72.33) | 260(71.82) | 0.846 |
| Bilateral nodules, n (%) |  | 847(58.29) | 208(57.46) | 0.773 |
| Bilateral focality, n (%) |  | 324(22.30) | 74(20.44) | 0.445 |
| Multifocality, n (%) |  | 457(31.45) | 105(29.01) | 0.368 |
| Capsular invasion, n (%) |  |  |  |  |
| Negative |  | 1243(85.55) | 314(86.74) | 0.818 |
| Proximity |  | 83(5.71) | 18(4.97) |  |
| Invasion |  | 127(8.74) | 30(8.29) |  |
| Capsular dorsal invasion, n (%) |  | 57(3.96) | 19(5.23) | 0.469 |
| Extrathyroidal extension, n (%) |  | 72(4.96) | 12(3.32) | 0.184 |
| Hashimoto's thyroiditis, n (%) |  | 227(15.62) | 55(15.19) | 0.84 |
| Abnormal LNs, n (%) |  | 1211(83.35) | 300(82.87) | 0.83 |
| LNs ultrasonic echo, n (%) |  |  |  |  |
| Hypoechoic |  | 1198(99.59) | 293(98.65) | 0.063 |
| Isoechoic |  | 5(0.42) | 4(1.35) |  |
| LNs size, mean (SD) (cm) |  | 1.55(0.85) | 1.49(0.74) | 0.27 |
| Unclear LNs border, n (%) |  | 70(5.98) | 20(6.92) | 0.551 |
| Irregular LNs morphology, n (%) |  | 246(16.94) | 60(16.53) | 0.124 |
| Abnormal lymphatic portal structure, n (%) |  | 167(11.50) | 42(11.57) | 0.271 |
| LNs microcalcification, n (%) |  | 500(41.29) | 129(43.00) | 0.59 |
| LNs vascularity, n (%) |  | 718(59.29) | 194(64.67) | 0.088 |
| **Pathological confirmation** |  |  |  |  |
| LLNM, n(%) |  | 915(62.97) | 220(60.77) | 0.439 |

^*^ means p-value < 0.05. ALT, alanine aminotransferase; AST, aspartate aminotransferase; APTT, activated partial thrombin time; BMI, body mass index; BUN, blood urea nitrogen; DBIL, direct bilirubin; DBP, diastolic blood pressure; FT3, free T3; FT4, free T4; HDL, high density lipoprotein; HGB, hemoglobin; LDL, low density lipoprotein; LLNM, lateral lymph node metastases; LNs, lymph nodes; MAP, mean arterial pressure; NLR, neutrophil-to-lymphocyte ratio; PLT, platelet; PT, prothrombin time; SBP, systolic blood pressure; SCR, serum creatinine; T3, triiodothyronine; T4, tetraiodothyronine; TBIL, total bilirubin; Tg-Ab, thyroglobulin antibody; TPO-Ab, thyroid peroxidase antibody; TSH, thyroid stimulating hormone; WBC, white blood cell count.
